# Histologic Evaluation of Thyroid Nodules Treated with Thermal Ablation: An Institutional Experience

**DOI:** 10.3390/ijms251810182

**Published:** 2024-09-22

**Authors:** Fernanda Russotto, Vincenzo Fiorentino, Cristina Pizzimenti, Marina Gloria Micali, Mariausilia Franchina, Ludovica Pepe, Giuseppe Riganati, Walter Giordano, Emilia Magliolo, Serenella Ristagno, Esther Diana Rossi, Giovanni Tuccari, Maurizio Martini, Antonio Ieni, Guido Fadda

**Affiliations:** 1 Department of Human Pathology in Adult and Developmental Age “Gaetano Barresi”, University of Messina, 98125 Messina, Italy; russottofernanda@gmail.com (F.R.); micalimarina@yahoo.it (M.G.M.); mariausilia.franchina@studenti.unime.it (M.F.); ludopepe97@gmail.com (L.P.); giuseppe.riganati@studenti.unime.it (G.R.); waltergiordano1997g@gmail.com (W.G.); giovanni.tuccari@unime.it (G.T.); maurizio.martini@unime.it (M.M.); antonio.ieni@unime.it (A.I.); guido.fadda@unime.it (G.F.); 2 Pathology Unit, Papardo Hospital, 98158 Messina, Italy; cristinapizzimenti86@gmail.com; 3 Department of Pathology, “San Vincenzo” Hospital, 98039 Taormina, Italy; emilia.magliolo@asp.messina.it; 4 Department of Oncology, Section of Endocrine Surgery, “San Vincenzo” Hospital, 98039 Taormina, Italy; serenellaristagno@gmail.com; 5 Division of Anatomic Pathology and Histology, Catholic University of Sacred Heart, Fondazione Policlinico Universitario A.Gemelli IRCCS, 00168 Rome, Italy; esther.rossi@policlinicogemelli.it

**Keywords:** thermal ablation, thyroid nodules, differential diagnosis, histological examination

## Abstract

Thyroid nodules are a common, benign condition with a higher prevalence in women, individuals with iodine deficiency, and radiation exposure. Treatment options for benign thyroid nodules include pharmaceutical therapy, thyroidectomy, and thermal ablation (TA). TA, including laser ablation (LA), radiofrequency ablation (RFA), and microwave ablation (MWA), is a procedure that uses heat to cause tissue necrosis. It is commonly used for large, firm, benign, non-functioning thyroid nodules that cause severe symptoms or pain when surgery is not recommended or desired. When thyroid nodules do not respond to TA, they undergo surgery to resolve the symptoms and clarify the diagnosis. This study aims to analyze the histological alterations found in surgically excised TA-treated thyroid nodules and to evaluate the morphological criteria of differential diagnosis between benign and malignant nodules, establishing whether the alterations observed on the histological sample are a consequence of TA or indicative of neoplastic disease. For this purpose, the adoption of ancillary methods, such as immunohistochemistry, is fundamental to distinguish the artifacts induced by TA from the typical morphological characteristics of malignant neoplasms.

## 1. Introduction

Nodular thyroid disease is a benign and quite common condition whose prevalence may vary depending on the population studied and the techniques used to detect nodules. Nodule incidence rises with age, and it is higher in women, in iodine-deficient individuals, and following radiation exposure. Numerous studies indicate a 2–6% prevalence with palpation, a 19–35% prevalence with ultrasonography, and an 8–65% prevalence in autopsy data [1]. The majority of benign thyroid nodules (BTNs) are asymptomatic or inactive. Large or multiple thyroid nodules may occasionally compress the esophagus and trachea, resulting in localized discomfort and swallow difficulty [2]. A pivotal role in the diagnosis of BTNs is played by fine-needle aspiration cytology (FNAC) under ultrasound guidance, and the treatment options include pharmaceutical therapy, which is seldom successful, thyroidectomy, and thermal ablation (TA) [3,4]. TA is a procedure that thermally removes the nodules, leaves no surgical scars on the neck, requires less time during surgery, and enables patients to quickly return to their normal lives [5]. In TA, the needle-electrode is introduced into the nodule under ultrasound guidance. High-frequency electromagnetic waves emitted by the electrode overheat the thyroid tissue, producing very localized necrosis of the nodule. The necrotic part will be partially reabsorbed and partially replaced over time by fibro-cicatricial tissue. The term TA refers to laser ablation (LA), radiofrequency ablation (RFA), and microwave ablation (MWA); they work by using heat to cause tissue necrosis [6]. Such techniques have recently been proposed and are currently utilized frequently. RFA is based on the creation of an electric field by a radiofrequency generator coupled to an electrode needle that is internally cooled, which, in turn, results in the production of heat. Moreover, MWA involves the use of a microwave generator that is capable of delivering 1–100 W of power at 2450 MHz in the form of a continuous flow or a pulse. When the water molecules in the abraded tissue oscillate, and the tissue is heated to cytotoxic levels, cell death results [7]. The percutaneous LA method is similar to RFA and MWA and uses a biopsy needle that is often inserted into the target nodules’ designated regions. The total energy provided during LA is normally between 1200 and 1800 J [8].

The indications for TA treatment are [9]:

- Large (volume > 20 mL), solid, benign, and non-functioning thyroid nodules that cause severe symptoms or pain when surgery is not advised or is not desired.
- Thyroid nodules with an autonomous function appear “hot” on scintigraphy when surgery or radioiodine treatment is not appropriate or desired.
- Local recurrence of thyroid cancer as palliative therapy when radioiodine is unsuccessful and surgery is not an option.

When thyroid nodules do not respond to TA, they undergo surgery in order to resolve symptomatology and/or to clarify the diagnosis with histopathological evaluation. In these cases, the potential pathological changes that TA could develop in the thyroid nodules might affect their correct histopathological diagnosis [6].

Moreover, ancillary techniques such as immunohistochemistry and molecular biology can be exploited in order to distinguish TA artifacts from malignant features in case of doubts. In fact, immunohistochemical markers such as galectin-3, HBME-1 (Hector Battifora’s “Mesothelioma” 1), BRAF V600E, and p53 have been shown to be helpful in the diagnostic phase of thyroid nodules, particularly in distinguishing benign from malignant lesions. Specifically, galectin-3 is a carbohydrate-binding protein that has been implicated in various cellular processes, including cell adhesion, proliferation, and apoptosis. In the context of thyroid nodules, its expression has been associated with malignant tumors, particularly papillary thyroid carcinoma and its variants [10]. Also, HBME-1, an antibody directed against mesothelin (an antigen typically found in mesothelial cells), is frequently used to diagnose thyroid carcinomas since its expression is often observed in malignant tumors, particularly papillary thyroid carcinoma and its follicular variant [11,12]. *BRAF* V600E, on its side, is a specific mutation in the *BRAF* gene that leads to the constitutive activation of the MAP kinase signaling pathway, and mutant BRAF V600E protein can be detected by immunohistochemistry. This mutation is strongly associated with papillary thyroid carcinoma, particularly the classic variant, and is considered a marker of aggressive tumor behavior and poorer prognosis [13]. Lastly, the p53 protein is a tumor suppressor that plays a critical role in regulating cell cycle progression and DNA repair. Mutations in the *TP53* gene can lead to the loss of its tumor suppressor function, contributing to the development and progression of various cancers, including thyroid carcinoma. The immunohistochemical overexpression of p53 can be a marker of p53 mutation and is associated with more aggressive tumor behavior and poorer prognosis in thyroid carcinoma [14].

The aim of our study was to identify specific histological characteristics of TA-treated thyroid nodules that can reliably differentiate them from thyroid carcinoma. By evaluating a panel of the four above-mentioned immunohistochemical markers, together with the proliferative index assessed with ki67, we aimed to improve the accuracy of histological diagnosis in distinguishing benign from malignant features.

## 2. Results

Patients’ characteristics and TA-treated nodules’ morphological features are reported in Table 1. The mean age of the patients was 56 years, with a prevalence of females (71.4%).

**Figure 1:**
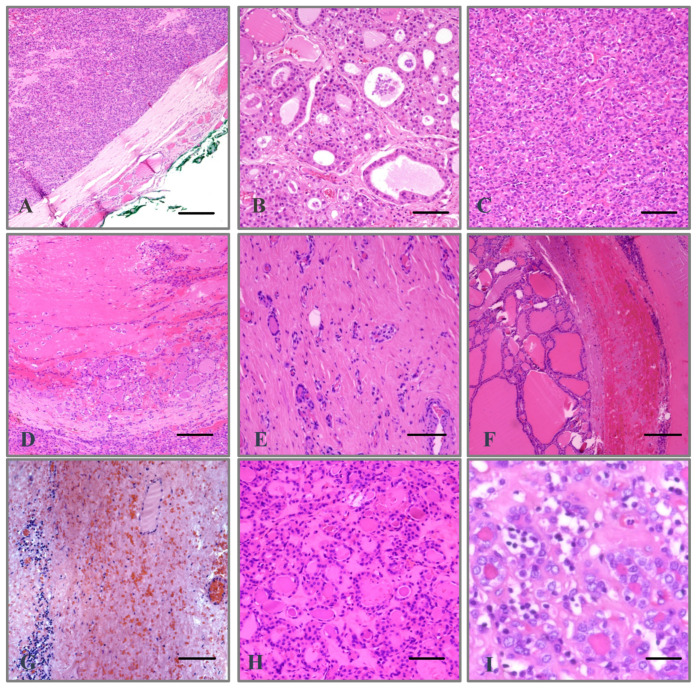
Histopathological findings of TA-treated thyroid nodules showing the presence of a fibrous capsule ((**A**), hematoxylin and eosin staining, 50× magnification; scale bar, 168 µm), follicular architecture ((**B**), hematoxylin and eosin staining, 100× magnification; scale bar, 84 µm), solid architecture ((**C**), hematoxylin and eosin staining, 100× magnification; scale bar, 84 µm), necrosis ((**D**), hematoxylin and eosin staining, 50× magnification; scale bar, 168 µm), sclerosis ((**E**), hematoxylin and eosin staining, 100× magnification; scale bar, 84 µm), hemorrhagic ((**F**), hematoxylin and eosin staining, 50× magnification; scale bar, 168 µm) and ischemic phenomena ((**G**), hematoxylin and eosin staining, 50× magnification; scale bar, 168 µm), dark ((**H**), hematoxylin and eosin staining, 100× magnification; scale bar, 84 µm) and clear nuclei ((**I**), hematoxylin and eosin staining, 200× magnification; scale bar, 42 µm). Oncocytic cells are visible at different magnifications in panels (**A**–**C**). TA: thermal ablation.

At histopathology, all nodules showed a capsule (Figure 1A), while a follicular pattern was evident in five out of seven (71.4%, Figure 1B) and a solid architecture in two out of seven (28.6%, Figure 1C). Moreover, TA can induce necrosis, which was observed in one out of seven nodules (14.3%, Figure 1D), whereas sclerosis was found in five out of seven cases (71.4%, Figure 1E). Additionally, hemorrhage was predominantly subcapsular and present in six out of seven nodules (85.7%, Figure 1F), whereas ischemic features were observed in one out of seven cases (14.3%, Figure 1G). Lastly, the nuclei were dark in four out of seven nodules (57.1%, Figure 1H) and clear in three out of seven nodules (42.9%, Figure 1I). Interestingly, a small proportion of oncocytic cells, varying from 10 to 20%, was seen in four out of seven nodules (57.1%, Figure 1A–C).

**Figure 2:**
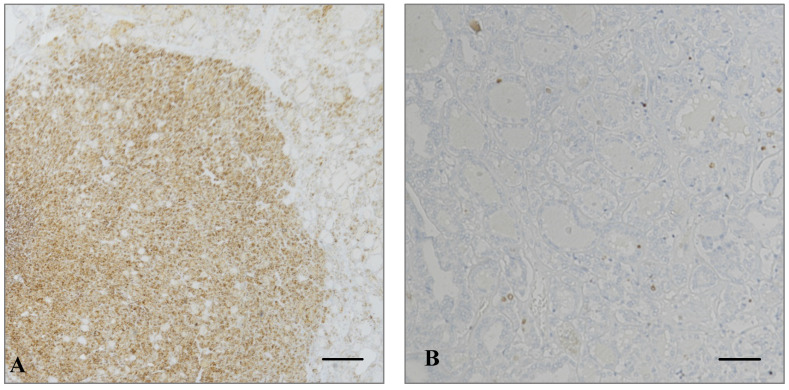
Immunohistochemical analysis of a focus of structural disarray in the only TA-treated nodule that tested positive for HBME-1 ((**A**), 100× magnification; scale bar, 84 µm). Ki-67 was less than 3% ((**B**), 200× magnification; scale bar, 42 µm). TA: thermal ablation.

At immunohistochemistry, six out of seven (85%) nodules were negative for HBME-1, and seven out of seven (100%) were negative for Gal-3, BRAF V600E, and p53. Of note, the only nodule that tested positive for HBME-1 showed immunoreactivity only in focal areas with structural disarray, including the presence of microfollicles and nuclear atypia, remaining negative in the other portions. Ki-67 Labeling Index (LI) was less than 3% in all cases (Figure 2 and Table 2).

## 3. Discussion

Patients with thyroid disease generally choose not to undergo surgery due to the risks associated with it. As a result, clinicians have started using minimally invasive methods to treat both benign and, more recently, malignant thyroid nodules (microcarcinoma or lymph node metastasis in patients with high surgical risks) [15]. Thyroid nodule volume reduction and cosmetic concerns, as well as the safety and efficacy of the treatment, have all been successfully addressed by TA of benign and malignant thyroid nodules. Symptomatology and cosmetic concerns brought on by nodular volume are the main reasons that induce treatment of benign thyroid nodules, and TA has been shown to be effective in facing these difficulties [2]. However, TA induces changes in treated tissues and TA-related histological changes regarding nodules’ architecture, nuclei features, the presence of a capsule, necrosis, sclerosis, hemorrhage, and ischemia. In our experience, TA-treated nodules showed a follicular or solid architecture and were surrounded by a fibrous capsule. Not surprisingly, sclerotic and necrotic areas potentially caused by TA were observed, and TA-related modifications of the nuclei and pseudo-capsular/vascular invasion could be identified. Moreover, hemorrhagic areas caused by passing the thermoablating needle along the thyroid parenchyma were noticed. In 2012, a study conducted at Arcispedale Santa Maria Nuova, Reggio Emilia (Italy), analyzing a case series of TA-treated nodules showed that the above-mentioned pathological findings were present in 22 patients over a period between 2 and 74 months from TA to the surgery. The basic histological characteristics of laser-induced tissue injury were present in all patients. These features comprised a well-defined region encircled by a fibrous capsule that held varying quantities of necrotic debris, inflammatory cells, and a fibrous scar. There were no notable pathologic characteristics discovered in the thyroid tissue close to the treated region, and the cytological features and follicular architecture were preserved. After a final histological analysis, none of the nodules that had received laser treatment turned out to be thyroid carcinomas [16]. Differently from our casuistry, necrosis was present in 10/12 patients, a significantly greater number than that observed by us (1/6 patients), and the prevalence of fibrosis was in 2/20 cases, while it was present in 5/7 patients in our series. Also, the aspects regarding hemorrhage, ischemia, and vascular and capsular invasion were not explored in depth, differently from our analysis (Table 3). Moreover, a recent study conducted at the Affiliated Hospital of Integrated Traditional Chinese and Western Medicine, Nanjing (University of Chinese Medicine, China) analyzed different pathological features of 39 TA-treated nodules over a time span of 6–12 months. Histopathological analyses revealed a high incidence of fibroblastic proliferation and chronic inflammation. Infarction, histiocytic deposition, blackish particles, foreign body reactions, cholesterol granulomas, necrosis, hemorrhage, and calcifications were the remaining pathological changes that had a high-to-low order of prevalence [17]. This study demonstrated that necrosis was present in 4/39 cases, a lower proportion of cases relative to our series (where it was present in 1/6 patients), and that fibrosis was evident in 32/39 cases, a higher proportion of cases relative to our series (where it was present in 5/7 patients). Hemorrhage was evident in 1/39 cases, a significantly lower proportion compared to our data (where it was detected in 1/6 cases), while infarction was more frequent in this study, being evident in 21/39 cases, differently from our study (where it was detected in 1/6 cases) (Table 3). Both studies allowed us to evaluate not only the histological aspects of TA-treated nodules but also the morphological criteria of differential diagnosis between benign and malignant thyroid nodules, assessing whether histological changes were the result of TA or suspicious for neoplastic pathology. In fact, some malignant lesions may share histological features with TA-treated nodules and possibly cause diagnostic problems. For example, papillary thyroid carcinomas can show sclerosing aspects; poorly differentiated carcinomas can contain areas of necrosis; or anaplastic (or undifferentiated) carcinomas may show areas of hemorrhage and necrosis [18]. Oncocytic neoplasms (also called oxyphilic or Hurtle cell neoplasms) may have areas of extensive necrosis and ischemic phenomena. In this respect, the presence of a significant proportion of oncocytic cells in TA-treated nodules may also influence the cytological preoperative diagnosis, causing overdiagnosis of oncocytic neoplasms. The features of the nuclei and the pseudo-capsular/vascular invasion serve as the primary determinants to distinguish a benign TA-treated nodule from a malignant one. Neoplastic nuclei (for example, in papillary thyroid carcinoma) have membrane irregularities and pseudo-inclusions, and cells are frequently crowded, are arranged in typical structures like papillae, and are often associated with microcalcifications. Instead, nuclei in TA-treated nodules are found near the TA needle and are surrounded by degenerative phenomena like fibrosclerosis [19]. Also, the pseudo-vascular invasion is distinguishable by the presence of epithelial cell fragments dispersed inside the vascular lumina, which often lack structure and exhibit clear signs of degeneration, possibly imitating fibrin. Conversely, signs of a true vascular invasion can be represented by the involvement of vessels within the capsule or outside the tumor itself. Moreover, true invaded venous or lymphatic vessels show a thin wall and a tumor-cast thrombus attached to the vessel intima, as well as lumen enlargement [20]. TA-induced artifacts can be associated with those related to the passage of the needle following an FNA procedure, in which a capsular rupture may occur along the path of the needle through the normal peripheral thyroid parenchyma, resulting in evidence of capsular pseudo-invasion [20,21]. Although they may look like extracapsular extensions, the fibrous tissue bands that encircle neoplastic nodules within a tumor really develop inward from the outer capsule, which normally retains a rather smooth exterior shape when intact. In follicular carcinoma, for example, the neoplastic follicular cells typically directly extend through the thyroid capsule and into the adjacent non-neoplastic thyroid tissue. Another possibility is the invasion of the capsule by tiny follicles that seem to percolate into the thyroid parenchyma [20,21]. Nonetheless, to the best of our knowledge, our study is the first in the literature to analyze LA-induced thyroid tissue changes in detail. However, while not discussing the presence of pseudo-capsular/vascular invasion, Piana et al. [16] and Cakir et al. [22,23] had already described the presence of an inflammatory infiltrate and fibrosis surrounding LA-treated nodules, which could lead to distortion and retraction of the normal tissue architecture, also involving the capsule and potentially creating the appearance of capsular invasion. Also, the authors described the presence of necrosis and inflammation in the ablated areas, which could lead to the disruption of vascular structures and the release of epithelial cell fragments into the vessels. The degenerative changes observed in these fragments could further complicate the distinction from fibrin. While these studies did not explicitly discuss pseudo-capsular/vascular invasion post-LA treatment, they described histological changes related to this procedure and, therefore, could provide a plausible explanation for our observations.

Finally, although our study focused on morphological aspects related to TA treatment, we wondered whether the residual vital ratio of thyroid nodules could represent a determining factor for benign nodular regrowth. In fact, recent literature data have shown that the residual vital ratio of thyroid nodules was the determinant factor for benign nodular regrowth [24]. Altogether, our results neither definitively confirm nor refute such findings: in fact, while the presence of some viable tissue is likely necessary for regrowth, the exact relationship between residual vital tissue and regrowth patterns remains to be fully elucidated. Further studies with quantitative assessment of residual vital tissue and long-term follow-up are needed to address this question comprehensively.

Overall, our study contributes to the understanding of the histological changes induced by TA in thyroid nodules, highlighting the importance of careful histopathological evaluation and the use of immunohistochemistry to differentiate between benign and malignant entities. Despite the novelty of our work and the scarcity of literature data regarding the histological evaluation of TA-treated thyroid nodules, our study presents some limitations, such as its small sample size and its retrospective nature, which may affect the generalizability of our results. Further research with larger cohorts and longer follow-up periods is therefore needed to validate our findings and evaluate the long-term effects of TA on thyroid nodules. In fact, some studies have reported that 4.1–37.5% of TA-treated thyroid nodules underwent regrowth after 2–3 years of ablation [24], and extending the follow-up period in future studies would provide more robust data on the durability of TA treatment and the incidence of late regrowth. Furthermore, our study focused on LA, and the results may not be generalizable to other TA techniques, such as RFA and MWA. Also, the absence of non-TA-treated control nodules limits the strength of our findings, and therefore, further studies with a larger sample size are needed to definitively differentiate between TA-induced artifacts and true neoplastic changes.

## 4. Materials and Methods

### 4.1. Case Selection and Morphological Features

In this study, we retrospectively reviewed 7 cases of thyroid nodules analyzed at the Pathological Anatomy Unit of the AOU Policlinico G. Martino (Messina, Italy) during a five-year period (January 2019 to December 2023).

For each case, the presence of a dominant nodule was assessed by ultrasound examination, then a fine-needle aspiration (FNA) was performed, and all nodules were found to be benign (according to SIAPEC 2014) [25]. Each patient underwent a TA treatment, and LA was used, an ultrasound-guided procedure that induces partial tissue necrosis and reduction of the volume of the nodule by inserting one or more 21G needles with optical fiber inside, providing energy laser. One to three needles were used. The total energy delivered during LA was between 1200 and 1800 J per fiber at each treated site. For each procedure, 2 sites were treated. The average reduction in nodule volume was 50% after 6 months. Patients with nodule regrowth after thermoablative therapy underwent surgery. Nodules subjected to this procedure should not exceed a volume of 20 mL and should have a diameter of less than 3 cm. The following total thyroidectomy was performed at the “San Vincenzo” Hospital in Taormina. Surgical specimens were fixed in 10% buffered formaldehyde, embedded in paraffin, cut to a thickness of five microns, and stained with hematoxylin–eosin. At histopathological examination, the following morphological features of each nodule were analyzed: architecture, nuclear characteristics, presence of a fibrous capsule, invasion, tissue and vascular necrosis, sclerosis, and hemorrhage. All patient data were collected anonymously, and written informed consent, as part of the routine diagnosis and treatment procedures, was obtained from patients or their guardians according to the Declaration of Helsinki, and the study adhered to Good Clinical Practice guidelines.

### 4.2. Immunohistochemical Analysis and Evaluation

For immunohistochemical procedures, 4-micron thick sections obtained from corresponding tissue blocks were deparaffinized, then washed in descending alcohol scale, treated with 3% hydrogen peroxide for 10 min, washed again in deionized water three times, and incubated with normal sheep serum to prevent unspecific adherence of serum proteins for 30 min at room temperature. Immunohistochemistry for HBME-1 (Dako, Glostrup, Denmark), galectin-3 (Gal-3, Ylem, Rome, Italy), Ki-67 (ThermoFisher Scientific, Waltham, MA, USA), BRAF V600E (ThermoFisher Scientific, USA) and p53 (ThermoFisher Scientific, USA) was carried out, according to the manufacturer’s instructions, using the labeled streptavidin–biotin–peroxidase complex system. The tissues were incubated with commercial monoclonal antibodies diluted 1:100 after prior microwave antigen retrieval (3 min passages in citrate buffer; pH 6.0).

The presence of clear nuclear features was defined as the detection of optically clear nuclei in more than 50% of the cells within a region of interest. Moreover, the definition of positivity for the tested immunohistochemical markers is as follows:

- HBME-1, cytoplasmic and membranous in more than 10 % of the lesional cells [26];
- Galectin-3, cytoplasmic, and nuclear in more than 10 % of the lesional cells [26];
- BRAF V600E, cytoplasmic in more than 30% of the lesional cells [27];
- p53, moderate (when visible with a 10× and/or 20× microscope objective lens) or strong (when visible with a 4× microscope objective lens) intensity in more than 30% of the nuclei of the lesional cells [14].

## 5. Conclusions

Overall, the novelty of our study is based on the application of an immunohistochemical panel made up of five antibodies (HBME-1, Gal-3, Ki-67, BRAF V600E, and p53) to detect malignant TA-treated thyroid nodules. Specifically, HBME-1 and Gal-3 are usually positive in thyroid carcinomas and negative in normal thyroid tissue. Similarly, BRAF V600E and p53 are the most commonly mutated genes in thyroid carcinomas, and antibodies against the corresponding mutated proteins can be used to identify neoplastic thyroid lesions. Lastly, Ki-67 provides an estimate of the proliferation of neoplastic cells. All the cases in our series tested negative for BRAF V600E, p53, HBME-1, Gal-3, and Ki-67, therefore excluding a cancer diagnosis. In conclusion, our findings suggest that immunohistochemistry may help attribute certain histological features to TA, potentially assisting in differentiating between TA-induced artifacts and neoplastic features. Although the negative immunohistochemical results for malignancy markers in the studied nodules suggest a TA-related etiology, further studies with larger sample sizes and the inclusion of non-A-treated nodules as positive controls would further strengthen our observations.

As a whole, our study provides valuable insights into the histological changes induced by TA in thyroid nodules detectable only by careful histopathological examination and proper immunohistochemical analysis and can help to improve the accuracy of diagnosis and management of patients with such lesions.

## Figures and Tables

**Table 1:** Patients’ characteristics and nodules’ morphological features.

	*N* = 7
Age, mean	56 years
Gender *n* (%)	
Male	2 (28.6%)
Female	5 (71.4%)
Architecture *n* (%)	
Follicular	5 (71.4%)
Solid	2 (28.6%)
Nuclei *n* (%)	
Dark	4 (57.1%)
Clear	3 (42.9%)
Number of nodules *n* (%)	
Single nodule	4 (57.1%)
Plurinodular	3 (42.9%)
Capsule *n* (%)	
Yes	6 (85.7%)
No	1 (14.3%)
Necrosis *n* (%)	
Yes	1 (14.3%)
No	6 (85.7%)
Fibrosis *n* (%)	
Yes	5 (71.4%)
No	2 (28.6%)
Hemorrhage *n* (%)	
Yes	6 (85.7%)
No	1 (14.3%)
Ischemia *n* (%)	
Yes	1 (14.3%)
No	6 (85.7%)
Capsular invasion *n* (%)	
Yes	0
No	7 (100%)
Vascular invasion *n* (%)	
Yes	0
No	7 (100%)
Presence of oncocytic cells	
Yes	4 (57.1%)
No	3 (42.9%)

**Table 2:** Immunochemical analysis of TA-treated nodules.

Markers	Results	Total *n*.7 (%)
Gal-3	Positive	0
	Negative	7 (100%)
HBME-1	Positive	1 (15%) *
	Negative	6 (85%)
p53	Positive	0
	Negative	7 (100%)
BRAF-V600E	Positive	0
	Negative	7 (100%)
MIB-1 (Ki-67)	<3%	7 (100%)

TA: thermal ablation; * only in foci with structural disarray.

**Table 3:** Main histopathological findings of TA-treated nodules in different studies.

Parameters	Current Study	Piana et al. (2012) [16]	Lu et al. (2021) [17]
	Patients 7	Patients 22	Patients 39
Age, mean	56 years	53 years	39 years
Gender *n* (%)			
Male	2 (28.6%)	3 (13.6%)	-
Female	5 (71.4%)	19 (86.4%)	
Architecture *n* (%)			
Follicular	5 (71.4%)	5 (22.7%)	-
Solid	2 (28.6%)	-	
Nuclei *n* (%)			
Dark	4 (57.1%)	-	-
Clear	3 (42.9%)		
Number of nodules *n* (%)			
Single nodule	4 (57.1%)	-	-
Plurinodular	3 (42.9%)		
Capsule *n* (%)			
Yes	6 (85.7%)	22 (100%)	-
No	1 (14.3%)	0	
Necrosis *n* (%)			
Yes	1 (14.3%)	10 (45.5%)	4 (10.26%)
No	6 (85.7%)	12 (54.5%)	35 (89.74%)
Fibrosis *n* (%)			
Yes	5 (71.4%)	20 (80%)	34 (87.18%)
No	2 (28.6%)	2 (20%)	5 (12.82%)
Hemorrhage *n* (%)			
Yes	6 (85.7%)	-	1 (2.56%)
No	1 (14.3%)		38 (97.44%)
Ischemia *n* (%)			
Yes	1 (14.3%)	-	21 (53.85%)
No	6 (85.7%)		18 (46.15%)
Capsular invasion *n* (%)			
Yes	0	-	-
No	7 (100%)		
Vascular invasion *n* (%)			
Yes	0	-	-
No	7 (100%)		
Presence of oncocytic cells *n* (%)			
Yes	4 (57.1%)	-	-
No	3 (42.9%)		

TA: thermal ablation.

## Data Availability

The original contributions presented in the study are included in the article, further inquiries can be directed to the corresponding author/s.
